# Reconstructing neuronal inputs from voltage recordings: application in the auditory system

**DOI:** 10.1186/1471-2202-12-S1-P14

**Published:** 2011-07-18

**Authors:** Stephen Odom, Christopher Leary, Gary Rose, Alla Borisyuk

**Affiliations:** 1 Department of Mathematics, University of Utah, Salt Lake City, UT 84112, USA; 2 Department of Biology, The University of Mississippi, University, MS 38677, USA; 3 Department of Biology, University of Utah, Salt Lake City, UT 84112, USA

## 

There are neurons in the auditory midbrain of frog *H. versicolor* that respond selectively to slowly rising auditory stimuli (behaviorally relevant for this frog). We investigate possible mechanisms that underlie this rise-time selectivity. In particular, we want to find out whether the rise-time selectivity arises in midbrain or is inherited from lower level structures.

To extract the relevant data from the existing recordings one needs to solve the inverse problem of computing the afferent inputs to the neuron. Reconstructing stimulus-evoked temporally-varying input to a neuron *in vivo* is a challenge. The existing model-based method allows us to resolve two synaptic conductances corresponding to two distinct reversal potentials. We present a new approach enabling the reconstruction of three input conductances. Our method is based on treating synaptic conductances and membrane voltage as random variables, generalizing the model to a stochastic differential equation, and deriving equations for both first and second moments. We apply reconstruction to simulated data and discuss applicability to experimental data.

Based on conductance reconstructions, we present three computational models of possible slow rise-time selectivity mechanisms: local inactivation of inhibition, fast-rise-time sensitive inhibition and interval counting with adaptation. We also discuss the evidence from in vivo recordings in support of the models. Finally, we show model predictions, which allow to distinguish between the proposed mechanisms as more data become available.

